# Danshen-Chuanxiong-Honghua Ameliorates Cerebral Impairment and Improves Spatial Cognitive Deficits after Transient Focal Ischemia and Identification of Active Compounds

**DOI:** 10.3389/fphar.2017.00452

**Published:** 2017-07-18

**Authors:** Xianhua Zhang, Wan Zheng, Tingrui Wang, Ping Ren, Fushun Wang, Xinliang Ma, Jian Wang, Xi Huang

**Affiliations:** ^1^Department of Clinical Pharmacology, Xiangya Hospital, Central South University Changsha, China; ^2^Institute of Clinical Pharmacology, Central South University, Hunan Key Laboratory of Pharmacogenetics Changsha, China; ^3^Institute of TCM-Related Comorbid Depression, Nanjing University of Chinese Medicine Nanjing, China; ^4^Department of Neurology, Binzhou Central Hospital, Binzhou Medical College Binzhou, China; ^5^School of Psychology, Nanjing University of Chinese Medicine Nanjing, China; ^6^Department of Emergency Medicine, Thomas Jefferson University, Philadelphia PA, United States; ^7^Department of Anesthesiology and Critical Care Medicine, School of Medicine, Johns Hopkins University, Baltimore MD, United States

**Keywords:** Danshen-Chuanxiong-Honghua (DCH), cerebral ischemia, inflammation, apoptosis, spatial cognitive function, neurogenesis

## Abstract

Previously, we only apply a traditional Chinese medicine (TCM) Danshen-Chuanxiong-Honghua (DCH) for cardioprotection via anti-inflammation in rats of acute myocardial infarction by occluding coronary artery. Presently, we select not only DCH but also its main absorbed compound ferulic acid (FA) for cerebra protection via similar action of mechanism above in animals of the transient middle cerebral artery occlusion (tMCAO). We investigated whether oral administration of DCH and FA could ameliorate MCAO-induced brain lesions in animals. By using liquid chromatography-tandem mass spectrometry (LC-MS/MS), we analyzed four compounds, including tanshinol, salvianolic acid B, hydroxysafflor yellow A and especially FA as the putative active components of DCH extract in the plasma, cerebrospinal fluid and injured hippocampus of rats with MCAO. In our study, it was assumed that FA played a similar neuroprotective role to DCH. We found that oral pretreatment with DCH (10 or 20 g/kg) and FA (100 mg/kg) improved neurological function and alleviated the infarct volume as well as brain edema in a dose-dependent manner. These changes were accompanied by improved ischemia-induced apoptosis and decreased the inflammatory response. Additionally, chronic treatment with DCH reversed MCAO-induced spatial cognitive deficits in a manner associated with enhanced neurogenesis and increased the expression of brain-derived neurotrophic factor in lesions of the hippocampus. These findings suggest that DCH has the ability to recover cognitive impairment and offer neuroprotection against cerebral ischemic injury via inhibiting microenvironmental inflammation and triggering of neurogenesis in the hippocampus. FA could be one of the potential active compounds.

## Introduction

Despite decades of research, ischemia stroke, which occurs due to the blood supply to brain becoming blocked, still remains one of the main causes of permanent disability and even death (Cheng et al., 2014; Nunez-Figueredo et al., 2014). Secondary damage from ischemia and reperfusion injury usually accompanies neuronal dysfunction and cell death, resulting in cognitive impairments and disability or even death. Without immediate medical treatment, a cascade of neuronal cells mostly located in the ischemic penumbra can die and undergo apoptosis quickly due to free radical overproduction and massive inflammatory responses (Xiong et al., 2013; Wang et al., 2017). Pro-inflammatory cytokines, especially IL-1β, can activate detrimental NF-κB nuclear translocation and increase p53-upregulated modulators of apoptosis, inducing neuronal apoptosis and structural brain injury (Shan et al., 2016).

Unfortunately, post-ischemic pro-inflammatory cytokines including tumor necrosis factor alpha (TNF-α) strongly degrade the microenvironment of nascent neurons, resulting in diverse forms of neuronal injury and low survival rates in the dentate gyrus (DG) of hippocampus (Ahmed et al., 2016). Additionally, low brain-derived neurotrophic factor (BDNF) protein levels in the hippocampus may contribute to deteriorative apoptosis of a subpopulation of hippocampal neurons, decreasing neuronal survival as well as impairing spatial learning and memory after cerebral ischemia (Fan et al., 2015; Soares et al., 2017). Despite advances in revealing the pathophysiology of cerebral ischemia at the molecular, cellular, and animal levels, the challenge of finding therapeutic options remains striking due to the serious side effects of drugs accompanied by a very short therapeutic window (≤6 h post-stroke) as well as secondary damage from ischemia and reperfusion injury (Donnan et al., 2008). Therefore, regulation of this post-ischemic inflammation and BDNF expression as well as endogenous hippocampal neurogenesis is a promising potential therapeutic strategy for restoring cognitive function that is impaired after ischemic insult.

Recently, interest in traditional herbal medicines has been growing, and traditional herbal medicines have become an important strategy for new drug development because their “multi-target” abilities, good synergy and “holistic” approach are more advantageous compared with “single target-single compound” approaches (Wang et al., 2012). The traditional Chinese formula Danshen-Chuanxiong-Honghua (DCH) is composed of *Salvia miltiorrhiza* Bge., *Ligusticum chuanxiong* Hort. and *Carthamus tinctorius* L. at a ratio of 2:1:1, which was optimized by Guanxin II (Huang et al., 2009). Widely used in Asia, especially in China, DCH is effective for treating ischemic heart diseases (IHD) (Qin et al., 2009) and providing anti-anginal effects (Abrams and Thadani, 2005). Meanwhile, it enhances anti-oxidative and anti-inflammatory responses and the coronary flow velocity (Qin et al., 2009; Zhang et al., 2010). Our previous study showed that DCH can prevent acute myocardial infarction (AMI) in rats by reducing the infarct size and suppressing myocardial cell apoptosis (Wang et al., 2011). Additionally, our pharmacokinetic study confirmed that seven compounds were absorbed into the blood following oral administration of DCH in rats (Zhang et al., 2017). On this basis, we will further explore which absorbed bioactive compounds (ABCs) were reached in injured brain, might be responsible for brain protection following oral DCH extract. In the current study, we investigated the neuroprotective effects of DCH pretreatment, with a particular focus on its anti-apoptotic and anti-inflammatory effects. On this basis, we also tested the hypothesis that DCH treatment promotes spatial cognitive function restoration after ischemic stroke by increasing neurogenesis and the BDNF level in hippocampus. In the present study, we used the middle cerebral artery occlusion (MACO) model to investigate the neuroprotective effects of DCH against acute cerebral ischemia and reperfusion injury in mice. Meanwhile, the Morris water maze (MWM) was used to assess the effects of DCH on rat brain function and endogenous hippocampal and neurogenesis neuroplasticity. As DCH has been widely used for thousands of years, it is safe and potentially beneficial for use in treating ischemic stroke.

## Materials and Methods

### Experimental Animals

All animal experimental procedures were implemented according to the institutional guidelines of the Animal Care and Use Committee of Central South University (Changsha, China). The protocol was approved by the animal experimental committee of Central South University. All efforts were made to minimize the pain and suffering of the animals. Male Kunming mice (25–28 g) and male Sprague-Dawley rats (250–270 g) were purchased from the Laboratory Animal Centre of Central South University. The animals were housed at a temperature of 23 ± 2°C with a 12/12 h dark/light cycle and free access to food and water. The animals were deprived of food for 12 h before the MCAO procedure was performed.

### Reagents

2,3,5-Triphenyltetrazolium chloride (TTC) and 5-bromo-2′-deoxyuridine (BrdU) were purchased from Sigma Chemical Co. Tanshinol, ferulic acid (FA), baicalin, protocatechuic acid, rosmarinic acid, salvianolic acid B, hydroxysafflor yellow A and 9′-methyl lithospermate B were purchased from Shanghai Yuanye Bio-Technology Co., Ltd. (Shanghai, China). Rabbit monoclonal Bax antibody (#14796s, 1:1000), rabbit monoclonal IL-1β antibody (#12426s, 1:1000), rabbit monoclonal IL-6 antibody (#12912p, 1:1000) were purchased from Cell Signal Technology. Mouse monoclonal TNF antibody (#60291-1-Ig, 1:500), rabbit polyclonal BDNF antibody (#25699-1-AP, 1:200), and mouse β-actin antibody (#60008-1-Ig, 1:4000) were purchased from Proteintech Group, Inc., and rabbit polyclonal Bcl-2 antibody (#bs-0032R, 1:100) was purchased from Bioss (Beijing).

### Preparation of DCH Extracts

The dry root and rhizome of *S. miltiorrhiza* Bge., the dry rhizome of *L. chuanxiong* Hort., and the dry flower of *C. tinctorius* L. were purchased from the pharmacy of Xiangya Hospital, Central South University and were authenticated by Professor SY Hu. The mixture of the DCH formula was soaked in distilled water (1:12, w/v) and then boiled twice for 30 min at 100°C. The twice-boiled blended supernatants were concentrated under pressure at 60°C and then lyophilized (yield = 30.53%, w/w), the product of which was sealed and stored at 4°C. The lyophilized powder was dissolved in saline to 2 g/mL before use.

### Experimental Groups and Administration of Drugs

**FIGURE 1:**
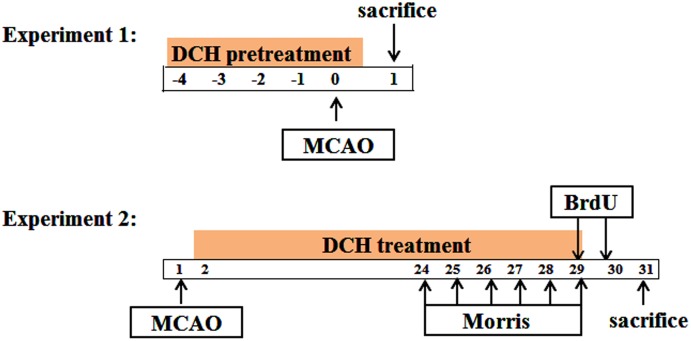
Schematic diagram of the experiment design. Time points represent days after MCAO.

The design of experiments is divided into two steps, as shown in **Figure 1**.

Part 1: DCH lyophilized powder or FA was dissolved with saline. To assess its preventive role in cerebral ischemia, DCH extracts (5, 10, and 20 g/kg) and FA (100 mg/kg) were oral administered once a day for five consecutive days before ischemia. Sham and vehicle mice were given equal volumes of saline via the same procedure.

Part 2: Beginning the day after MCAO, DCH extracts (20 g/kg) and FA (100 mg/kg) were oral administered once a day for 28 consecutive days. Similarly, sham and vehicle mice were administered an equal volume of saline via the same procedure.

### Preparation of the Focal Cerebral Ischemia and Reperfusion Model

Transient focal cerebral ischemia was induced by MCAO, followed by reperfusion as previously reported (Longa et al., 1989; Zhou et al., 2012) with minor modifications. In brief, after mice were anesthetized with an intraperitoneal injection of chloral hydrate (400 mg/kg), a midline neck surgical incision was made, and the ipsilateral external carotid artery (ECA) was carefully separated and ligated. A 6.0 nylon monofilament suture (Beijing Cinontech Co., Ltd, Beijing, China) coated with 1% poly-L-lysine was inserted from the right common carotid artery (CCA) to the internal carotid artery (ICA) and gently advanced to occlude the origin of the middle cerebral artery (MCA) at the knot of the Circle of Willis. The suture was gently removed after 90 min of occlusion to allow reperfusion. An identical surgery was performed on sham-operated animals, except that the intraluminal suture was withdrawn immediately to allow prompt reperfusion. The animals were housed in a warm cage for approximate 2 h to maintain their body temperature after the operation. Similarly, the rat preparation of focal cerebral ischemia was the same as in mice, except that a 4.0 nylon monofilament suture was inserted into the right MCA and removed after 120 min of occlusion.

### Neurological Deficit Assessment

The neurological score was evaluated by an observer blinded to the animal groups at 24 h after reperfusion as described previously (Bederson et al., 1986). The neurologic score was specified as follows: 0, no deficit; 1, flexion of contralateral forelimb; 2, decrease of resistance toward the contralateral plane; 3, circling monolaterally; 4, unable or difficult to ambulate.

### Evaluation of Infarct Volume and Ipsilateral Edema

After assessment of neurological deficit, mice were killed by decapitation with anesthesia. The brains were rapidly removed and frozen at -20°C for 10 min and then sliced into 2-mm-thick coronal sections. The coronal sections were incubated in 2% TTC in phosphate-buffered saline (pH 7.4) at 37°C for 30 min in the dark and then stored in a 4% paraformaldehyde solution overnight at room temperature. The infarct area was morphometrically measured using Image Tool 2.0 software (University of Texas Health Science Center, Texas, TX, United States). The total infarct volume for each brain was calculated by adding the lesion areas of all brain sections (infarct area × thickness [2 mm]) from the same hemisphere. Correction for ipsilateral edema was accomplished by comparing the volume of the ipsilateral and contralateral hemispheres as previously described (Lin et al., 1993). The percentage increase of the ischemic hemisphere volume was expressed as (ipsilateral volume – contralateral volume)/contralateral volume × 100%.

### Morris Water Maze Test

The Morris water maze is widely used as an assessment of spatial learning and memory (Morris et al., 1982), and it was applied in this study from day 24 to 29 after MCAO (**Figure 1**). In brief, the water maze device consisted of a circular water tank (120 cm in diameter and 50 cm in depth) in a dark and quiet room with several prominent visual cues. The water temperature was maintained at 25 ± 1°C. A black rounded platform (10 cm in diameter) was submerged 2.0 cm below the surface of the water in the center of the quadrant and stayed in the same position during the training period. Rats’ swimming trajectories were recorded by a video camera with a computer, and the parameters were sent to an image analyzer.

Each rat was trained twice per day for five consecutive days. For every trail, the rat was placed into the water facing the wall of the pool from four starting points in a different order and allowed to swim. The time to reach the hidden platform (escape latency) was measured for up to 90 s. If the rat failed, it would be guided to find the platform and allowed to remain there for 15 s, and their escape latency was recorded as 90 s. Finally, 24 h after the last training day, the platform was removed and the rats were tested on a 90-s retention probe trial. The number of times the rats crossed the position where the platform had previously been located was also recorded during the 90-s trail.

### Immunofluorescence

For the cellular proliferation study, BrdU (Sigma), 50 mg/kg in saline was intraperitoneally injected twice daily at 8-h intervals on consecutive days (**Figure 1**). To examine the number of newly formed cells in the DG of the injured hippocampus, the animals were perfused transcardially with ice-cold saline, followed by 4% paraformaldehyde in phosphate buffer (PB, 0.1 M, pH 7.4) 12 h after the last BrdU injection (during day 27 and 28 after MCAO) (Jin et al., 2001). Afterward, the brains were dehydrated in 30% sucrose in phosphate buffer for 24 h and fixed overnight in paraformaldehyde for 24 h and then were cryoprotected. Serial coronal sections (40 μm) were cut on a sliding freezing microtome (Leica, Germany) and collected in PBS.

Every fifth coronal brain section throughout the hippocampus, which was 400 μm apart, was selected for BrdU immunofluorescence staining as previously described (Yang et al., 2015a). In brief, sections were pretreated with 2 N HCl at 37°C for 60 min followed by rinses in 0.1 M borate buffer for 10 min (pH 8.5). Sections were incubated with PBS (0.5% Triton X-100, 10% normal goat serum) for 1 h at room temperature and then incubated in rat monoclonal anti-BrdU (1:400; Abcam, Cambridge, United Kingdom) at 4°C overnight. After being washed three times with PBS, sections were incubated for 2 h with FITC conjugated goat anti-rat IgG (1:200, Proteintech Group, Inc.) at room temperature. Brain sections were washed again and submerged in DAPI solution (1:1000) for 10 min, then cover slipped.

Newborn cells were authenticated by co-labeling with BrdU-positive cells and DAPI. BrdU-positive cells in DG of injured hemisphere were counted and analyzed in a blinded fashion in each section under fluorescence confocal microscopy (Leica SP8, Germany). The results are expressed as the total number of cells per DG, which was obtained by calculating the total number of BrdU-positive cells per section and multiplying by 10.

### Western Blot Analysis

Mice (part 1: at post-ischemic 24 h) or rats (part 2: at post-ischemic day 28) were decapitated under anesthesia, the hippocampus was separated from the lesion hemisphere, frozen quickly in liquid nitrogen, and stored at -80°C. The hippocampus tissues were disrupted in PBS and homogenized with RIPA buffer by ice-cold Immune Precipitation Assay and then centrifuged at 12000 × *g* for 15 min. Protein samples of 50 μg per lane were separated on polyacrylamide gel and then transferred onto a polyvinylidene difluoride (PVDF) membrane. Membranes were blocked with a 5% milk solution (non-fat dry milk in TBST) for 1 h and then incubated with specific antibodies as described above at 4°C overnight. Following five washes with TBST for 15 min each, the membranes were incubated with the corresponding HRP conjugated goat anti-mouse IgG (1:4000, Proteintech Group) or HRP conjugated goat anti-rabbit IgG (1:6000, Proteintech Group) for 1 h at room temperature. Immunoreactivity was detected with a Pierce^TM^ ECL substrate (Thermo) for 3 min, and then, the samples were exposed to *X*-ray films. The values were normalized to the intensity levels of β-actin and analyzed using Quantity-One software (Bio-Rad, Hercules, CA, United States).

### Preparation of Biological Sample after Oral Administration of DCH to Rats

Six rats were subjected to MCAO. A small amount of plasma and CSF was collected at 60 min after oral administration of DCH extract (20 g/kg) and then sacrificed under anesthesia. Subsequently, the hippocampus tissue was separated rapidly from the injured hemispheres on the ice tray and stored at -80°C. All samples (approximately 50.0 mg) were homogenized in 1.5 mL of ice-cold 0.9% saline, prepared per our previous description. Briefly, biological sample were extracted separately with ethyl acetate and methanol using the liquid–liquid extraction (LLE) method, and the two supernatants were combined and dried with nitrogen, then reconstituted in 100 μL of 70% methanol and centrifuged at 13000 rpm for 10 min. A 15 μL aliquot of the supernatant was injected into the liquid chromatography-tandem mass spectrometry (LC-MS/MS) system for qualitative analysis. Active phytochemicals from the DCH extract in biological samples were identified by comparing the retention time in the LC-MS/MS method with an 300SB-C18 column (4.6 mm × 250 mm, i.d., 5 μm, Agilent, United States) according to our previous experiment. In brief, HPLC was performed using an Agilent Technologies 1200 series LC system (Agilent Corporation, Santa Clara, CA, United States), and an ABI 3200Q-TRAP quadrupole linear ion trap mass spectrometer was operated. The mobile phase was composed of 0.1% formic acid (A) and acetonitrile (B) using a gradient elution as follows: 15% B from 0 to 3 min; 15–50% B from 3 to 8 min; 50% B from 8 to 10 min; 50–75% B from 10 to 11 min; 75% B from 11 to 14 min; 75–5% B from 14 to 15 min; and 5% B from 15 to 17 min. The flow rate was set at 1 mL/min, and the injection volume was 15 μL. The electrospray ion source (ESI) was used in the negative ion mode, employing MRM with the precursor-to-production pairs during this experiment. The procedure was controlled by Analyst 1.4.1 data acquisition and processing software (applied Biosystems/MDS Sciex).

Reference standard solution of each of the following compounds was prepared directly in methanol or water: tanshinol, FA, baicalin, protocatechuic acid, rosmarinic acid, salvianolic acid B, 9′-methyl lithospermate B, and hydroxysafflor yellow A. Calibrated standard stock solutions of gradient concentrations containing the eight constituents were serially diluted with a mixture of methanol/water (70:30, v/v). The contents of these components in the DCH extract were calculated by using the regression parameters acquired from the standard curves.

### Statistical Analysis

All data are presented as the mean ± SEM. The escape latencies in the Morris water maze test were compared using two-way analysis of variance (ANOVA) with repeated measure. The other data were evaluated by one-way ANOVA. Significance levels were expressed as a value of *p* < 0.05. Statistical analysis was performed using GraphPad Prism 5 software.

## Results

### Chromatographic Analysis of Active Components in DCH Extracts

**FIGURE 2:**
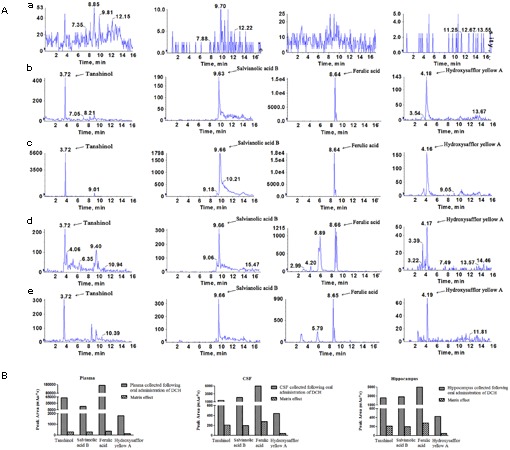
Identification of the active compound of DCH extract in the plasma, CSF and injured hippocampus of rats subjected to MCAO. Representative multiple reaction monitoring (MRM) chromatograms of tanshinol, FA, salvianolic acid B and hydroxysafflor yellow A were exhibited **(A)**. Blank plasma **(a)**; Blank plasma spiked with the four compounds **(b)**; Plasma sample **(c)**; CSF **(d)** and injured hippocampal tissue **(e)** were collected at 0.5 h after a single oral administration of DCH extract (20 g/kg). The presence of the four compounds in plasma, CSF and hippocampus was showed in bar graphs **(B)**.

To calculate the administered dose, a chromatographic analysis of DCH extract showed that the concentrations of tanshinol, FA, baicalin, protocatechuic acid, rosmarinic acid, salvianolic acid B, 9′-methyl lithospermate B and hydroxysafflor yellow A were 0.743, 0.115, 0.005, 0.011, 0.391, 3.631, 0.009, and 3.109 mg/g, respectively. Moreover, to explore what active compounds were absorbed in blood and brain, which exerted potentially preventative and therapeutic effect on cerebral ischemia, we qualitatively analyzed using LC/MS-MS. Biological samples of rats pretreated with DCH extract revealed that four peaks were present in blood, CSF, and hippocampus of the injured hemisphere of rats. As shown in **Figure 2A**, their retention times were 3.72, 9.66, 8.65, and 4.17 min, respectively. To identify the active compounds in the biological samples of DCH-pretreated rats, we performed the ESI and MRM with their precursor-to-production pairs and compared the retention of the four selective standards based on our previous study. The four peaks were tanshinol, salvianolic acid B, FA, and hydroxysafflor yellow A, which had retention times closest to that of biological samples of rats. Additionally, FA had the strongest response (**Figure 2**), which likely indicates that its concentration is the highest in target tissue among the four active compounds. Therefore, FA was selected to test similar preventative effect and underlying mechanisms of DCH on cerebral ischemia in our study.

### DCH Pretreatment Reduces MCAO-Induced Infarct Mouse Neuronal Injury

**FIGURE 3:**
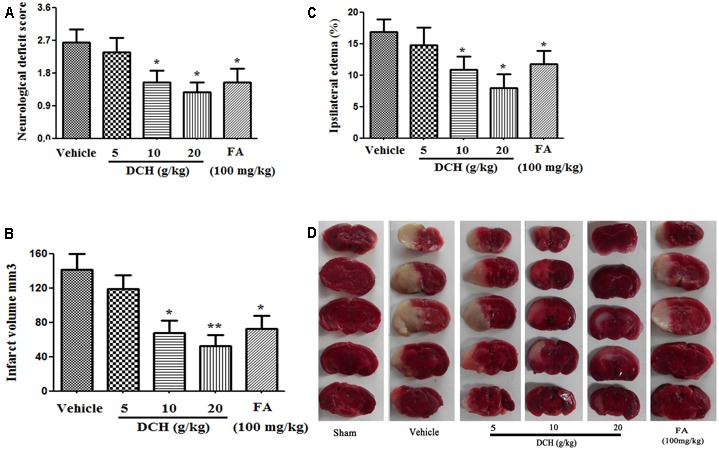
Preventive effects of oral DCH on brain injury after focal cerebral ischemia in mice. DCH (5, 10, or 20 g/kg) and FA (100 mg/kg) were orally administered for five consecutive days before inducing brain ischemia. The neurological deficit score **(A)**, infarct volume **(B)**, and ipsilateral edema **(C)** were determined. **(D)**, Representative brain coronal sections (2 mm thick) stained with 2% TTC. Typical infarct regions are indicated in white and normal regions are indicated in red. Data are represented as mean ± SEM, *n* = 10, ^∗^*p* < 0.05, ^∗∗^*p* < 0.01 versus vehicle group.

To assess the potential preventative effects of DCH extract on mice subjected to MCAO, mice were pretreated with DCH extract (5, 10, or 20 g/kg/day) for five consecutive days before the induction of cerebral ischemia. The neurological scores were evaluated 24 h after MCAO. As shown in **Figure 3A**, the neurological scores were significantly attenuated in DCH extract (10, 20 g/kg)- and FA (100 mg/kg)-pretreated mice compared with those in a vehicle-treated mice, whereas no significant neurological dysfunction was observed in the sham group. Moreover, staining of brain sections with TTC revealed that pretreatment with DCH extract (10, 20 g/kg) and FA (100 mg/kg) greatly alleviated the total infarct volume (**Figure 3B**) and brain edema (**Figure 3C**) compared with the vehicle-pretreated group. These results suggested that DCH and FA pretreatment improved the functional outcome after cerebral ischemia and reperfusion injury (**Figure 3**). As a control, mice in the sham group did not show neurological dysfunction or cerebral infarction (data not shown).

### DCH Pretreatment Alleviates Stroke-Induced Inflammatory Reaction and Apoptosis

**FIGURE 4:**
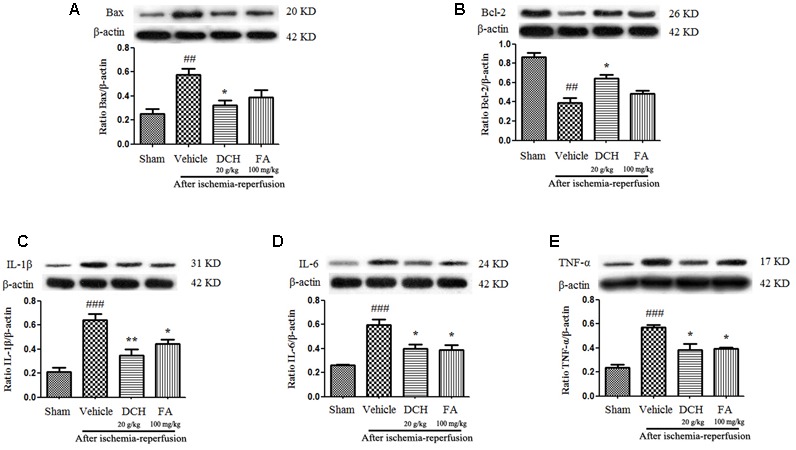
Danshen-Chuanxiong-Honghua extract alleviated apoptosis and inhibited pro-inflammatory cytokine production in the injured hippocampus 24 h after cerebral ischemia and reperfusion. Protein levels of Bax **(A)**, Bcl-2 **(B)**, IL-1β **(C)**, IL-6 **(D)**, and TNF-α **(E)** were measured by western blot. Quantified results were normalized to β-actin expression. Data are expressed as mean ± SEM, *n* = 4, ^###^*p* < 0.001, ^##^*p* < 0.01 versus sham group; ^∗^*p* < 0.05, ^∗∗^*p* < 0.01 versus vehicle group.

We found significantly enhanced protein levels of IL-1β, IL-6, TNF-α, and Bax, and reduced the levels of Bcl-2 24 h after MACO in injured hippocampus compared with sham-operated group by western blot (**Figure 4**), all which were significantly reversed by DCH pretreatment (**Figure 4**). Together, these results showed that DCH pretreatment mitigated the inflammatory response and apoptosis in the injured hippocampus of mice. However, FA pretreatment only ameliorated the levels of inflammatory cytokines (**Figure 4**).

### DCH Improved Ischemia-Induced Spatial Cognitive Deficits

**FIGURE 5:**
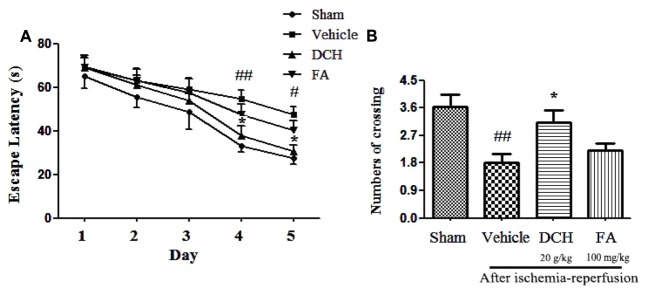
Danshen-Chuanxiong-Honghua improved the cognitive deficits induced by ischemic stroke in rats. DCH reversed the prolonged escape latency in vehicle group **(A)**. The number of crossing was recorded on the 6th day **(B)**. Data were expressed as mean ± SEM, *n* = 10–13, ^##^*p* < 0.01, ^#^*p* < 0.05 versus sham group; ^∗^*p* < 0.05 versus vehicle group.

To investigate the effect of chronic DCH and FA treatment on cognitive function, we exposed the animal to the Morris water maze test after 28 days of treatment. All rats exhibited a progressively shorter escape latency in a day-dependent manner by training every day, and there was a obviously differences among groups (**Figure 5A**). Two-way ANOVA showed that, beginning on day 4, animals in the sham group demonstrated a shorter latency to finding the platform compared with those in the vehicle group (*p* < 0.05), and the results were similar on day 5 (*p* < 0.05). These data clearly show the impairment of memory by experiment-induced focal cerebral ischemia. The escape latency was greatly decreased on day 4 (*p* < 0.05) and day 5 (*p* < 0.05) after DCH treatment. Although it was not statistically significant, FA treatment also rendered shorter escape latencies than the vehicle group (*p* > 0.05). These results indicated that DCH treatment effectively ameliorated spatial learning through 5-day training. As shown in **Figure 5B**, in the probe test without the platform, vehicle-rats demonstrated a lower the number of times crossing the platform area compared to rats in the sham group (*p* < 0.05). Compared with vehicle group, DCH-treated rats crossed the platform location more frequently (*p* < 0.05). Unfortunately, there was no statistically significant discrepancy in the FA group (*p* > 0.05). Therefore, DCH treatment for 28 days obviously improved ischemia-induced spatial memory impairments (**Figure 5**).

### DCH Increased the Neuroproliferation

**FIGURE 6:**
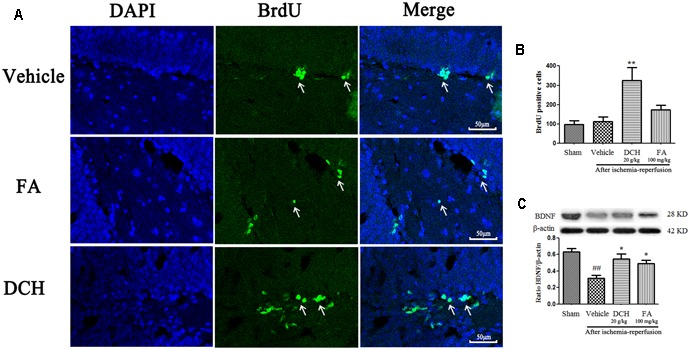
Danshen-Chuanxiong-Honghua increased neurogenesis and the expressions of BDNF. Representative confocal microscopy images were immunolabeled with BrdU positive cells (green) **(A)**. Quantitative analysis of BrdU positive cells **(B)**. Protein levels of BDNF were measured by western blot **(C)**. Quantified results were normalized to β-actin expression. Data were represented as mean ± SEM, *n* = 6–8, ^##^*p* < 0.01 versus sham group; ^∗∗^*p* < 0.01, ^∗^*p* < 0.05 versus vehicle group.

Ischemic lesions induce neurogenesis in the DG, and newborn cells reach a peak at post-ischemic day 7 and then die (Yang et al., 2015b). Interestingly, compared with the sham-operated group, newborn cells returned to the original standard in the DG of the damaged hemisphere 28 days after surgery. However, BrdU positive cells were approximately threefold higher after DCH treatment on day 28 after MCAO compared with the vehicle group (*p* < 0.01, **Figures 6A,B**). BrdU positive cells were increased less than twice in the FA group (*p* > 0.05, **Figures 6A,B**). This result indicated that DCH promotes basic neurogenesis and strengthens inherent neurogenic processes.

### DCH Pretreatment Increases Expression of BDNF Protein

Brain-derived neurotrophic factor is used as a indicator of neuroplasticity and considered to be involved in neurogenesis. As shown in **Figure 6C**, expression of BDNF protein was found to be markedly decreased in the lesions hippocampus on day 28 after MCAO compared with the sham group. Oppositely, DCH treatment greatly increased the BDNF level compared with the vehicle group.

## Discussion

Stroke is a leading factor that is responsible for death and long-term disability worldwide, imposing a substantial burden on patients, their relatives, and entire economies (Kim and Johnston, 2011). In particular, large hemispheric infarctions commences with the occlusion of the cerebral vasculature and deprivation of oxygen and nutrient support to specific regions of the brain, subsequently causing regional brain damage and functional outcomes that can lead to severe morbidity and mortality (Ma et al., 2013). Despite the use of thrombolytic drugs during acute ischemic stroke, narrow therapeutic time-window and risk of intracranial hemorrhage significantly hamper their administration in clinic practice (Merino-Zamorano et al., 2015). Currently, none of available drugs have been shown to be effective in clinical trials of ischemic stroke (Jin et al., 2013). Therefore, the search for promising and safe neuroprotective agents is particularly urgent and important. Here, we find that DCH usually focuses on a “holistic” approach and multiple targets to improve the neuroprotective effect and functional outcomes. As DCH is safe to use based on thousands of years of experience in East Asia (Qin and Huang, 2009), it may be a potential therapeutic strategy for ischemia stroke. A previous study indicated that DCH protects the heart again oxidative stress, inflammation, and cell apoptosis induced by acute ischemic myocardial injury in rats (Qin and Huang, 2009). However, there is no direct evidence proving the neuroprotective effects of DCH in ischemic stroke.

Strikingly, four components, particularly FA, were simultaneously absorbed into blood, CSF and hippocampus of rats subjected to MCAO following oral administration of DCH extract (**Figure 2**). Surprisingly, the content of FA was far lower than that of the other ingredients in DCH extract, but its peak area was approximately threefold stronger compared with the other in the target tissue (**Figure 2B**). It seems to confirm that FA has great oral absorption capacity and blood-brain barrier permeability, attributed to its favorable physicochemical properties. Intriguingly, it is reported that FA plays a neuroprotective role against oxidative Neuro-2a cells, possibly by inhibiting hydrogen peroxide induced oxidative stress and down-regulating nNOS, COX-2, and IL-1 (Dong et al., 2015). It also can prevent neuronal cell death against cerebral ischemic injury, thought the MEK/ERK/p90RSK/Bad signaling pathway (Koh, 2015). Therefore, these findings support the possibility that FA is the main active component of DCH, which is representative of its multiple biomechanism. In our study, DCH and FA pretreatment decreased neurologic deficit and the cerebral injury volume (**Figure 3**) as well as reduced the protein levels of IL-1β, IL-6, and TNF-α in the hippocampus of the injured hemisphere after MCAO (**Figure 4**). Simultaneously, DCH pretreatment also suppressed ischemia-induced apoptosis possibly through inhibition of inflammatory responses due to reduced secretion of IL-1β, IL-6, and TNF-α (**Figure 4**). In addition, the results indicated that DCH treatment for 28 days improved cognitive function by promoting neurogenesis and expression of BDNF protein in DG after cerebral ischemia and reperfusion (**Figures 5, 6**). It was further demonstrated that FA is the active compound of DCH and exerts similar neuroprotective effects and is part of the underlying mechanism of the effect of DCH on ischemic stroke.

Inflammation has been increasingly considered to play a critical role in the pathophysiology of post-stroke lesions (Liesz and Kleinschnitz, 2015). During acute ischemic stroke, sterile inflammation induces the generation of pro-inflammatory cytokines, such as IL-1β, IL-6, and TNF-α, which successively accelerate the neuroinflammatory response (Barrington et al., 2017). Ischemic injury induces neuronal death and the release of damage associated molecular patterns (DAMPs). The pro-inflammatory cytokines IL-1β and TNF-α have been implicated as key contributors to ischemic stroke and reperfusion (Murray et al., 2015). IL-6, a multifunctional cytokine, has an excessive inflammatory response, which might augment injury due to acute ischemic stroke (Jin et al., 2013; Yang et al., 2016). Furthermore, previous studies have exhibited that brain IL-1β and TNF-α levels increase within a few hours and peak at 24 h after MCAO (Jin et al., 2013), whereas IL-6 concentrations increase from 24 h up to 7 days. Here, these pro-inflammatory cytokines (IL-1β, IL-6, and TNF-α) were chosen as markers to evaluate the inhibition of the inflammatory response at 24 h after cerebral ischemia and reperfusion in the hippocampus. All of the above evidence demonstrates that elevated levels of IL-1β, IL-6, and TNF-α may play a critical role in inflammation-induced neuronal death after acute stroke. Interestingly, our findings indicate that DCH pretreatment inhibits inflammatory responses and improve neuronal repair by reducing the secretion IL-1β and IL-6 as well as TNF-α in acute ischemic stroke (**Figure 4**). As we all know, apoptosis is another pivotal role in the survival of cells after ischemic damage (Pan et al., 2016). Bax, an accelerator of apoptosis, is activated by releasing of cytochrome C from the mitochondria. Oppositely, Bcl-2 inhibits the apoptosis and neuronal death (Deng et al., 2017). In addition, a few studies have reported that pro-inflammatory cytokines induce NF-κB nuclear translocation and enhancing P53-upregulated modulator of apoptosis (PUMA); then, p53 is released from Bcl-X_L_ by activated PUMA, which induces Bax activation and results in apoptosis within the infarcted hemisphere, ultimately leading to irreversible brain insult (Shan et al., 2016). In line with these previous studies, our results suggested that the protective effect of DCH against neuronal apoptosis was associated with inhibiting inflammatory responses, which decreased the secretion of IL-1β, IL-6, and TNF-α, leading to downregulation of Bax and the upregulation of Bcl-2 in the hippocampus of mice in the acute phase of ischemic stroke (**Figure 4**).

In addition to its acute neuroprotective effects on reducing infarct size, secretion of inflammatory cytokines, and inhibition of apoptosis, we also investigated the robust neuroprotective effects of DCH during the chronic phase of stroke subjected to transient MCAO. The impairment of spatial memory tasks is generally considered to be induced by ischemia and reperfusion insults in the hippocampus (Yang et al., 2015a), and we thereby used the MWM to survey cognitive deficits. Interestingly, our results demonstrated that DCH-treatment for 4 weeks significantly reduced the escape latency during the training compared with that of vehicle treatment, suggesting that DCH decreased the ischemia-induced spatial learning deficits. In the same way, DCH also attenuated the spatial memory deficits, which were exposed by the time spent in the target quadrant and the number of rats crossing the platform position in the probe trail. These results indicated that DCH treatment for 4 weeks alleviated learning and memory impairment induced by MCAO.

The course of learning and memory is closely relevant to the hippocampus (Yang et al., 2015a). Thus, delayed neuronal death in the hippocampus induced by MCAO results in neurological deficits, such as cognitive impairment. In the present study, we examined the effects of DCH treatment for 4 weeks on the recovery phase of cerebral ischemia, especially hippocampal neurogenesis. Previous studies have suggested that the cerebral ischemia-induced neurogenesis was increased in the rodent subgranular zone (SGZ) of DG of the injured hemisphere in adult rodents (Kang et al., 2013; Yang et al., 2015a). Hippocampal neurogenesis exerts a crucial role in functional outcome and promotes spatial memory recovery (Liu et al., 2007). Therefore, long-lasting promotion of neurogenesis in the hippocampus after a stroke might be a new strategy for the alleviation of post-stroke cognitive impairment. BrdU, a thymidine analog, is incorporated into cellular DNA during the S phase of cell proliferation and thus has been widely used to examine cell proliferation (Yang et al., 2015b). Here, we found that DCH treatment for 4 weeks significantly increased the number of Brdu positive cells in the DG after MCAO, indicating that DCH might improve the proliferation of stroke-induced newborn cells, and the majority of these newborn cells matured into neurons in the DG. Mature hippocampal neurons are involved in learning and long-term memory formation (Suarez-Pereira et al., 2015). Thus, our results indicate hippocampal neurogenesis may play a beneficial role in DCH-induced spatial cognitive amelioration after ischemic stroke.

To further study the mechanism of DCH on enhancing cognitive outcomes and hippocampal neurogenesis, we analyzed the levels of pro-inflammatory cytokines as well as BDNF protein in the ischemic hippocampus after DCH pre-treatment or treatment. Neuroinflammation begins to disrupt the regulation of neurogenesis, causing irreversible damage to neurons in the ischemic core, even in the ischemic penumbra after ischemic stroke (Liu et al., 2007; Chapman et al., 2015). Accumulation of pro-inflammatory cytokines immensely alters the microenvironment of the neural stem cells, which is deleterious to progenitor survival and proliferation (Das and Basu, 2008; Ahmed et al., 2016). In support of this notion, this neuroinflammation-induced unfavorable microenvironment was reversed by DCH treatment, contributing to the promotion of neurogenesis after ischemic stroke. We found that the plentiful secretion of pro-inflammatory cytokines, including IL-1β, IL-6, and TNF-α caused by ischemic and reperfusion was significantly inhibited by DCH pretreatment. Taken together, the mechanism of DCH-increased proliferation of newborn cells is, in part, associated with its anti-inflammatory effects after stroke. BDNF is an crucial neurotrophin factor that is involved in memory formation and storage (Bekinschtein et al., 2007). As a modulator of neurogenesis, BDNF plays important roles in neurogenesis and neuroplasticity, as well as in the self-rescue of different types of neurons after ischemic stroke (Pang et al., 2004; Venna et al., 2014). Additionally, BDNF also improves neurogenesis and accelerates the migration of new neurons in the DG (Schabitz et al., 2007). Here, our results suggested that BDNF levels in the hippocampus were markedly enhanced by DCH treatment. In view of the role of BDNF in neurogenesis and neuroplasticity and that DCH increases the expression of BDNF, we conclude that DCH treatment might depend on upregulating BDNF expression to facilitate hippocampal neurogenesis.

In summary, with anti-inflammatory, anti-apoptotic and neuroprotective effects, DCH might represent a promising prevention and therapy for ischemic stroke. Moreover, our results demonstrate that chronic DCH treatment ameliorates spatial cognitive impairment and promotes neurogenesis as well as neuroplasticity in the lesions hippocampus. We speculate that the attenuation of inflammation and enhancement of neuroplasticity are necessary for the effect of DCH on neurogenesis, resulting in improving spatial cognitive function after MCAO. The active component FA via inhibiting inflammation and increasing the BDNF level (**Figures 4, 6**) might be, in part, responsible for the neuroprotective effects of DCH in ischemic stroke.

## Author Contributions

XH and PR are in charge of funds, protocol, experiment and accomplishment with the help of XZ, WZ, TW, JW, XM, and FW. XZ wrote the manuscript and XH correct it. XZ performed chromatographic analysis. XZ and TW carried out animal experiments. XZ and WZ analyzed experimental results and plotted graphs and pictures.

## Conflict of Interest Statement

The authors declare that the research was conducted in the absence of any commercial or financial relationships that could be construed as a potential conflict of interest.
